# Gastric outlet obstruction in a patient

**DOI:** 10.1002/emp2.13285

**Published:** 2024-10-20

**Authors:** Dragan Vasin, Miona Jevtovic, Sabina Fiuljanin, Katarina Trajković, Tarik Plojović, Marković Danilo, Dušan Micić, Ksenija Mijovic, Aleksandar Pavlović, Dragan Mašulović

**Affiliations:** ^1^ Center for Radiology University Clinical Center of Serbia Belgrade Serbia; ^2^ Faculty of Medicine University of Belgrade Belgrade Serbia; ^3^ General Hospital Novi Pazar Novi Pazar Serbia; ^4^ Clinic for Emergency Surgery University Clinical Center of Serbia Belgrade Serbia

## 1 CASE HISTORY

An 81‐year‐old man with a history of hypertension presented to the emergency department with epigastric pain, vomiting, hiccups, anorexia, and obstipation for 3 days. Physical examination was notable for a painful epigastric tenderness. Laboratory examinations revealed a white blood cell count of 22.1 (3.4–9.7)(10 × 9/L).

**FIGURE 1:**
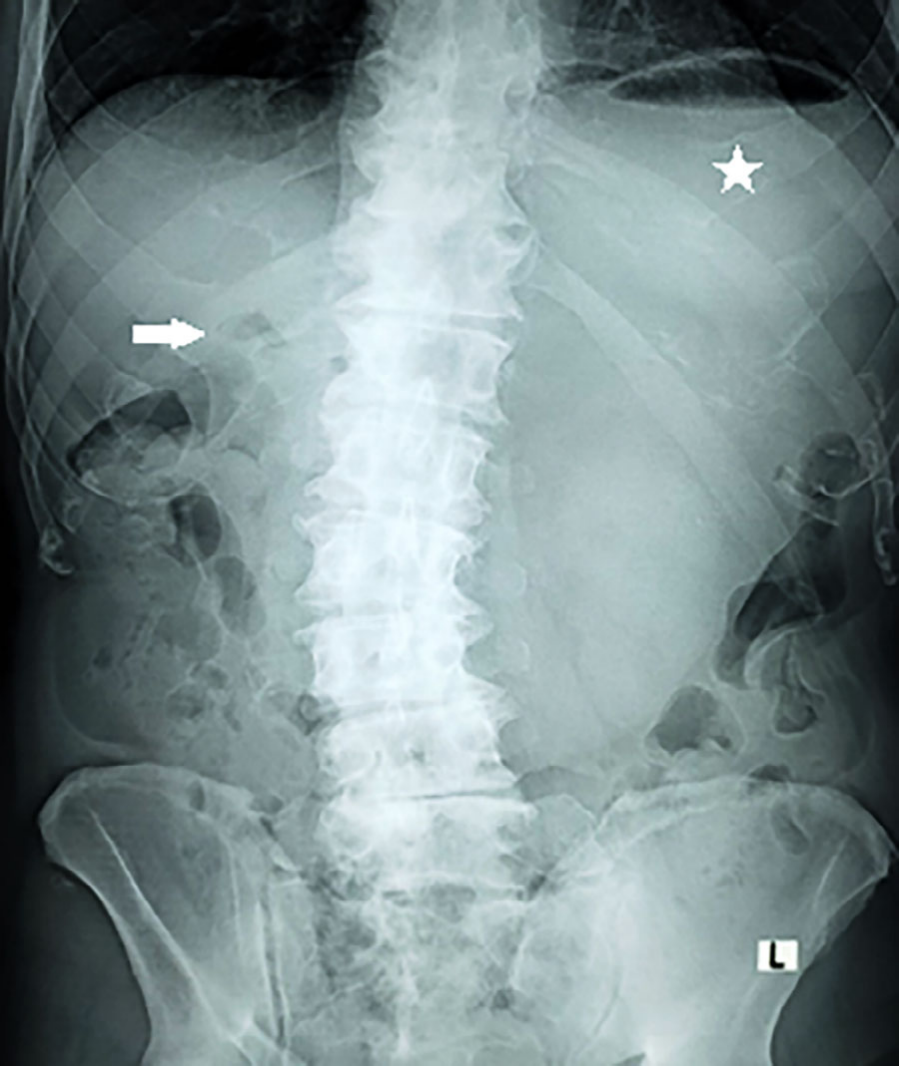
Plain abdominal radiography demonstrating pneumobilia (arrow) within the right upper quadrant and enlarged stomach (star).

**FIGURE 2:**
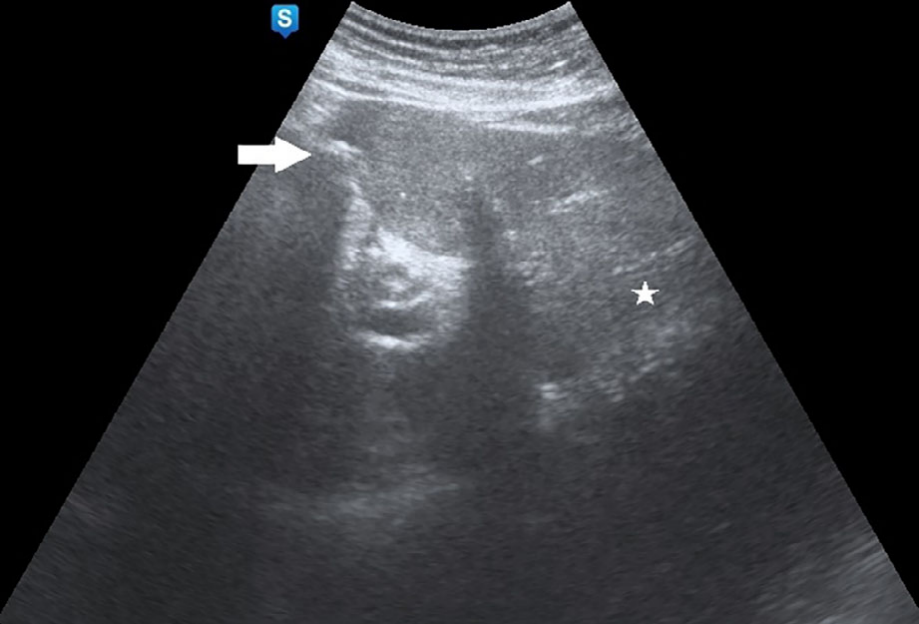
Transverse sonogram of the expected location of the duodenal bulb shows a curvilinear focus of increased echogenicity with posterior shadowing (arrow), findings consistent with a large gallstone with multiple foci of hyperechogenicity in the left liver lobe with ring‐down artifact (star), which suggests pneumobilia.

**FIGURE 3:**
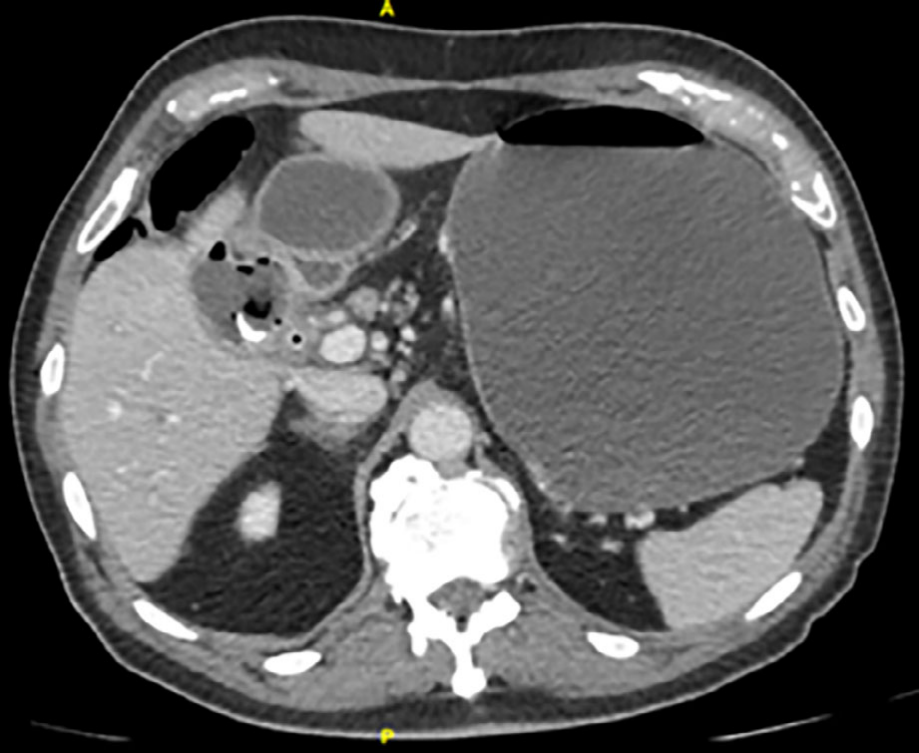
Axial image from abdominal computed tomography shows a calcified structure in the duodenal bulb and distended stomach.

Plain abdominal radiography showed pneumobilia and an enlarged gastric bubble (Figure 1), and abdominal ultrasound also demonstrated an enlarged stomach with a large amount of content within a curvilinear focus of increased echogenicity with posterior shadowing in duodenal bulb (Figure 2). Subsequent computed tomography (CT) image is shown in Figure 3.

## 2 DIAGNOSIS

### 2.1 Bouveret's syndrome

The images observed from the patient's CT are typical of Bouveret's syndrome. It is characterized by gastric outlet obstruction (GOO), secondary to the impaction of a large gallstone through an acquired fistula between the gallbladder and either the duodenum or stomach.^1^ It is named after the French internist Léon Bouveret who published two case reports of this condition in the Revue Medicale in 1896.^2^ A rare complication of cholelithiasis, biliodigestive fistulae occur in less than 1% of all patients. Fistula formation is favored by the long history of cholelithiasis, the repeated episodes of acute cholecystitis, the large size of the gallstones (2–8 cm), the female gender, and advanced age (>60 years). Morbidity and mortality rates are high, estimated at 60% and 12%–30%, respectively, due to the advanced age and the comorbidities of the patients.^3^ CT is the best imaging technique used to search for Bouveret's syndrome with its 93% sensitivity, 100% specificity, and 99% accuracy needed for definitive diagnosis.^4^

## CONFLICT OF INTEREST STATEMENT

The authors declare no conflicts of interest.

## Data Availability

Raw data were generated at University Clinical Center of Serbia. Derived data supporting the findings of this study are available from the corresponding author on request.
